# Cancer-Related Health and Educational Needs and Faith-Based Health Beliefs in an Urban Muslim Population

**DOI:** 10.1007/s13187-024-02439-4

**Published:** 2024-04-23

**Authors:** Aisha Choudhri, Lucile Adams-Campbell, Mireille Bright, Jialing Zhu, Chiranjeev Dash

**Affiliations:** Ralph Lauren Center for Cancer Prevention at Georgetown University’s Lombardi Comprehensive Cancer Center, Washington, D.C USA

## Abstract

Cancer screening behaviors in Muslims are under-researched, and there is limited data on how it relates to their unique cultural and religious beliefs. We assessed cancer prevention and screening-related health needs in the Washington DC area. We developed the needs assessment questionnaires and recruitment strategy in collaboration with key faith leaders from four mosques in our catchment area. A total of 203 participants were recruited through community outreach and engagement approaches and were included in the discussion when developing the needs assessment to ensure questions were religiously and culturally sensitive. Of the 203 participants, 56% of women reported receiving screening for a mammogram, while 83% of women reported receiving a screening for cervical cancer. Among men, 45% reported receiving a prostate cancer antigen test to screen for prostate cancer. Among both men and women, 35% reported ever receiving a screening for colorectal cancer. Women reported relying more on their faith when dealing with health concerns than men. Those who did not get screened for breast, colorectal, and cervical cancer relied more on their faith than those who did get screened for these cancers. Participants expressed interest in having health initiatives around cancer education, screening, and survivorship inside mosques. Faith beliefs can influence cancer screening behaviors; however, the relationship between these two variables needs further examination. Continued engagement with key faith leaders can help in leveraging religious beliefs to promote health education and cancer screening.

## Introduction

There are an estimated 3.4 million Muslims in the USA, and this population is expected to dramatically increase in the next few decades. Specifically, the Washington D.C. metropolitan area has one of the largest Muslim communities in the country, ranking number three in most observed faiths in the region [1, 2]. U.S. Muslims are diverse in race and ethnicity, with no single racial group constituting the majority population. More (41%) Muslims in the USA identify as White (including Arabs and people of Middle Eastern descent) than Asian (28%; including people of Pakistani or Indian descent) or Black or African American (20%) [2]. While cancer data based specifically on religious affiliation is not routinely collected in the USA, cancer rates based on racial identification are documented. Whites (including those of Arab descent), South Asians, and Black or African Americans comprise the largest racial groups among Muslims in the USA, and these racial groups have some of the highest incidence and mortality rates of cancer [3]. While being one of the fastest growing and racially and ethnically diverse religions in the USA, healthcare disparities among American Muslims remain under-investigated, and there are limited studies that look at the cancer-related health needs of this population.

Muslims often share common religious beliefs. These beliefs create a framework for how many American Muslims may view their health through a religious lens and how these religious accommodations may influence decisions concerning their health. Among beliefs are concepts of modesty, gender-concordant preferences and availability of healthcare providers, reliance on faith components and religious scripture for healing, and beliefs of fatalism when faced with serious health concerns. Religious practices, such as prayer, were believed to play a role in health promotion, prevention of serious illness, providing a cure, and the first preventive course of action when dealing with individual health concerns [4]. Ritual prayers, supplication, and reading of the Qur’an (the Islamic holy scripture) can be used as primary sources of healing or used to complement traditional, allopathic treatment [5, 6]. Modesty among Muslims is important when dealing with cross-gender interactions, and this concept is attributed to Islamic teachings around gender relations and dress codes. These values in turn may influence health care choices for Muslims and present them with challenges during clinical encounters [4, 7, 8]. Specifically, studies have suggested that these values may prevent Muslims from obtaining breast, cervical, and colorectal cancer screenings [9–15].

As suggested in a systematic literature review, there is a paucity of research on the cancer-related health needs of this community, specifically regarding cancer screening practices [16]. Moreover, how Islamic beliefs and values inform Muslim health-related behaviors is relatively under-investigated across different cancers [16]. To the best of our knowledge, no Muslim needs assessments have been conducted in the Washington D.C. metropolitan area, specifically looking at cancer screening practices, as well as the role of spirituality and faith in making health decisions. Thus, we sought to provide data on the cancer-related health needs of Muslims residing in the Washington, D.C. metropolitan area, where some of the largest populations of Muslims reside within the USA by conducting a needs assessment survey [17]. Additionally, we sought to better understand how certain faith components inform cancer screening and preventive health practices. Understanding the spiritual and religious underpinnings that drive or inhibit certain health-seeking behaviors would allow for tailoring cancer outreach and education to better serve the Muslim community.

## Methods

### Study Design, Population, and Partnerships

This study is a cross-sectional survey among a convenient sample of Muslims. Prior to the development of the needs assessment survey, we identified four mosques (places of worship for Muslims), based on their locations within the D.C., Maryland, and Virginia areas. One mosque was located in D.C., one in Maryland, and two mosques were located in Virginia. We initiated contact with and established relationships with the key members of these mosques who serve as spiritual and administrative leaders. We introduced the rationale and scope of our needs assessment. We engaged in dialogue with these leaders and applied their input about connections that can be made between components of the Islamic faith and health concepts and healing to ensure that the needs assessment survey was culturally and religiously sensitive.

### Cultural Competency Training

Imams and other faith leaders expressed the need for staff to be aware of certain guidelines such as gender boundaries and the etiquette of entering the mosque when approaching congregants to take the survey. Other concerns consisted of making sure that questions on the needs assessment survey did not force participants to reveal any personally identifiable information and to be sure that questions that were asked did not go against the tenets of the Islamic faith. Based on this feedback, all office staff received cultural competency training on interacting with Muslim populations, regardless of study involvement. Components of the cultural competency training included defining the basic tenets of the Islamic faith, describing stigmas Muslims may hold regarding their health and seeking healthcare, the role of the imam in promoting health, dietary restrictions, gender boundaries, beliefs of fatalism, and privacy concerns. The goal of the training was to enhance staff knowledge and understanding of participants’ potential beliefs.

#### Study Measures

The needs assessment questionnaire consisted of 43 items. Qualtrics was used to develop and electronically distribute the needs assessment survey with the following components: demographics, access to care and cancer-related health needs, cancer screening compliance and practices, family and personal health history, and the role of religion and spirituality in preventive health behaviors. Muslims taking the survey were asked three faith-based questions: (1) When you are sick, do you rely on natural/cultural remedies instead of going to see a doctor?, (2) do you seek guidance from an imam or spiritual advisor when dealing with a major health issue(s), and (3) as a Muslim, do you rely on ritual prayers, supplication, and reading of the Qur’an as primary sources of healing a health issue or concern?

### Participant Eligibility and Recruitment

To be eligible for participation, participants had to be aged 18 years or older, able to consent to taking the survey, and able to read and write in English. Two study coordinators worked on distributing the survey. Study coordinators obtained verbal consent, before handing out paper copies or providing the QR code to the survey, without any further interaction with the participants. Participants could complete the survey either electronically or using a paper version. To help limit the chance of multiple responses from the same person, once the respondent was determined to have completed the survey on their phone or computer, the respondent was sent out of the survey and an end of survey message was displayed, which prevented the respondent from using the same anonymous link again on their device. Additionally, our data manager looked for duplicate surveys by checking the I.P. address of the survey entries. The survey was provided in English. Recruitment strategies included visits to mosques during Friday (Jummah) prayers to distribute study flyers and questionnaires, requesting imams to make announcements about the study after Jummah prayers, and having Mosque administration send information about the study and the survey link through email, as well as posting these materials on their website. Additionally, health fairs held at mosques were attended by members of the staff to distribute the survey.

### Data Analysis

Demographic characteristics are reported as means (standard deviation) for continuous variables and count (frequency) for categorical variables. Conventional sociodemographic items such as age, gender, race/ethnicity, educational background, and health insurance status were considered to be potential explanatory variables for health behaviors. Descriptive statistics to summarize study measures were calculated using SAS (Statistical Analysis Software). Significant differences were determined by the chi-square test for categorical variables and Fisher’s exact tests for count data. A *p*-value < 0.05 was considered to be statistically significant. “Prefer not to answer” responses were combined with “no” responses, and “yes” responses were combined with “sometimes” responses for faith-based categorical variables. When asking cancer screening questions on the needs assessment survey, we based eligibility to be screened from screening recommendations provided by the American Cancer Society for breast, cervical, prostate and colorectal cancer. Cancer screening data obtained from the needs assessment was compared to cancer screening rates reported in the District of Columbia’s Cancer Control Plan [18], the Virginia Cancer Plan [19], and the Maryland Comprehensive Cancer Control Plan [20]. Screening data obtained from Washington D.C., Virginia, and Maryland’s cancer control plan was collected from individuals eligible for cancer screenings for breast, cervical, prostate, and colorectal cancer based off of guidelines from the American Cancer Society, National Cancer Institute, the National Comprehensive Cancer Network (NCCN), the United States Preventive Services Task Force, (USPSTF), and data collected from the Behavioral Risk Factor Surveillance System (BRFSS).

## Results

### Demographic Characteristics

A total of 203 participants completed the needs assessment survey. Summary characteristics are presented in Table 1. The majority of those surveyed were female (64.5%). The mean age of respondents was 48.9 years with 78% of the participants ≥ 35 years of age and 40% of participants self-reported as Asian, while 30.6% identified as Black/African American. Most participants reported having a Bachelor’s or advanced degree, and 52.5% of respondents reported English as their primary language, while 27.8% reported Urdu/Hindi/Punjabi, which is specific to the South Asian population, as their primary language (Table 1). The majority (79%) of participants reported having access to primary care. Of those who had access to care, 88% reported visiting their primary care physician for a routine checkup or blood work in the past 12 months. When asked where they regularly receive this care, participants most often (59%) reported receiving it with a primary care physician in a private office. A total of 17% of Muslims reported ever smoking at least 100 cigarettes in their life, and the majority (95%) of smokers reported not currently smoking. The participants’ mean time spent doing moderate-intensity physical activity, fitness, or leisure activities on a typical day was 38 min.

**Table 1:** Baseline characteristics of participants surveyed

Categories	Characteristics	Mean ± SD or *N* (%)
Gender	Male Female	52 (33.3) 100 (64.5)
Age (SD)		48.9 ± 17.3
Education	High school diploma or less Some college Bachelor’s/advanced degree	10 (6.8) 30 (20.4) 107 (72.7)
Race/ethnicity	Black/African American Asian White/Caucasian Multi-race Spanish/Hispanic/Latino Other	45 (30.6) 59 (40.1) 15 (10.2) 12 (8.1) 6 (4.0) 16 (10.8)
Primary language	English Urdu/Hindi/Punjabi Arabic Other	140 (52.6) 74 (27.8) 18 (6.7) 34 (12.7)
Access to primary care	*Yes* *No*	151 (79) 36 (19)

*+ Row numbers may not add up to total n as “prefer not to say” or unreported not included*

## Cancer Screening Among Participants

Mammography uptake was reported by 56% of women. Among those who had ever received a mammogram, 50% of women reported receiving a mammogram less than 12 months ago, while 25% reported receiving one within the past 2 years. Additionally, 83% reported receiving a cervical cancer screening through either a Pap test or an HPV test. Among men, 45% received a prostate-specific antigen (PSA) blood test to screen for prostate cancer. Among both men and women, 35% of respondents reported ever receiving a colonoscopy to screen for colorectal cancer (Table 2).

**Table 2:** Distribution of cancer screening among the study population and screening eligible adults in the Georgetown Lombardi Catchment area

Cancer screening	Muslim population *N* (%)	Washington, D.C (%)	Virginia (%)	Maryland (%)
Mammogram	54 (56%)	80%	76.2%	81.2%
Pap or HPV test	83 (83%)	82%	79.8% (PAP) 51.8% (HPV)	81.3%
PSA test	23 (45%)	39%	46.6%	55.7%
Colonoscopy	51 (35%)	82%	67.6%	72.5%

### Cancer Screening Rates in the Georgetown Lombardi Catchment Area

We compared the cancer screening percentages reported in the needs assessment to our survey respondent area of Washington. D.C., Maryland, and Virginia. When we compare the overall cancer screening rates to the Washington, D.C. area, the percentage of women reporting receiving a mammogram was 80%, compared to 56% of Muslim women. The rates of cervical cancer screening were similar for D.C. (82%) and Muslims (83%). Muslims had a higher percentage of screening for prostate cancer (45%) when compared to those in Washington, D.C. (39%). In regards to colorectal cancer screening, Muslims were well below (35%) the Washington D.C. screening rates of 82%. When we compare Muslim cancer screening percentages to Virginia, the rates for mammograms (76.2%), PSA testing (46.6%), and colonoscopy (67.6%) were higher in Virginia residents compared to percentages reported in the needs assessment. The screening rates for Pap and HPV testing in Virginia were reported to be 79.8% and 51.8%, respectively. In Maryland, the rates of mammograms (81.2%), PSA testing (55.7%), and colonoscopy(72.5%) were higher in Maryland residents when compared to Muslims who reported receiving these cancer screenings. The rates of cervical cancer screening were similar in Maryland (81.3%) and those reported among the Muslim population (83%) [18–20] (Table 2).

### Faith Beliefs and Health Behaviors

Women reported relying more on all three faith components compared to men with a significant gender difference in relying on natural and cultural remedies instead of going to see a doctor (Table 3). Differences in the faith components based on the two most self-identified races reported in the needs assessment were also noted. Asian Muslims relied on natural and cultural remedies (63.5%) and ritual prayers, supplication, and scripture as primary sources of healing (58.7%) more than Black/African Americans (36.5% and 41.2%, respectively). Regarding seeking guidance from an imam or spiritual advisor when dealing with health issues, Black/African American Muslims (51.7%) relied on this faith component more than Asian Muslims (48.3%).

**Table 3:** Reliance on faith components by gender

Faith component	Total number of respondents	Female *n* (%)	Male *n *(%)	* p*-value
Rely on natural/cultural remedies instead of going to see a doctor	150	74 (76%)	23 (23%)	< 0.01
Seek guidance with an imam or spiritual advisor when dealing with health issues	148	28 (62%)	17 (38%)	0.42
Rely on ritual prayers, supplication, and reading of the Quran as primary sources of healing	149	78 (68%)	37 (32%)	0.33

*Table only includes data from affirmative responses

Table 4 demonstrates reliance on faith components among participants who reported whether they had ever received screening for breast, cervical, prostate, or colorectal cancer. Among the women who reported receiving a mammogram, 72% relied on natural and cultural remedies instead of going to see a doctor, 22% sought guidance from an imam or spiritual advisor when dealing with a major health issue, and 75% relied on ritual prayers, supplication, and Quran as primary sources of healing. Among the women who reported receiving a cervical cancer screening, 74% relied on natural and cultural remedies instead of going to see a doctor, 24% sought guidance with an imam or spiritual advisor when dealing with a major health issue, and 79% relied on ritual prayers, supplication, and Quran as primary sources of healing.

**Table 4:** Reliance on faith components among participants who reported whether they had ever received screening for breast, cervical, prostate, and colorectal cancer

	Screened for breast cancer		Screened for cervical cancer		Screened for prostate cancer		Screened for colorectal cancer	
Faith components	Yes (*n* = 54)	No (*n* = 42)	Yes (*n* = 83)	No (*n* = 17)	Yes (*n* = 23)	No (*n* = 26)	Yes (*n* = 51)	No (*n* = 93)
Rely on natural/cultural remedies instead of going to see a doctor	72%	76%	74%	88%	30%	57% *p* = 0.05	54%	68%
Seek guidance from an imam or spiritual advisor when dealing with health issues	22%	34%	24%	50%	50% *p* < 0.05	19%	30%	29%
Rely on ritual prayers, supplication, and reading the Quran as a primary source of healing.	75%	82%	79%	80%	65%	77%	68%	80%

When looking at men and prostate cancer screening, 30% of those who were screened for prostate cancer relied on natural and cultural remedies instead of going to see a doctor (*p* = 0.05), 50% sought guidance with an imam or spiritual advisor when dealing with a major health issue (*p* < 0.05), and 65% relied on ritual prayers, supplication, and Quran as primary sources of healing. For both men and women, among the 36% who reported receiving a colonoscopy, 54% relied on natural and cultural remedies instead of going to see a doctor, 30% sought guidance with an imam or spiritual advisor when dealing with a major health issue, and 68% relied on ritual prayers, supplication, and Quran as primary sources of healing (Table 4).

### Mosque-Based Health Initiatives

Participants reported desiring various health initiatives inside their mosques (Table 5). The most commonly desired initiatives were nutrition counseling (25.5%), physical activity (25.3%), cancer education/screening (15.6%), and cancer treatment/survivorship (10.2%). A small proportion of participants self-reported desired health initiatives (5.45%), and most of these participants listed mental health services as their desired health initiative.

**Table 5:** Health initiatives desired inside mosques by participants

Health initiative	% of participants
Nutrition counseling	25.5
Physical activity	25.3
Cancer education/screening	15.6
Cancer treatment/survivorship	10.2
Genetic counseling	9.3
Smoking cessation Other	8.8 5.4

## Discussion

In one of the first cancer prevention and screening-related needs assessments among Muslims in the Washington, D.C. metropolitan area, we report an overall lower rate of cancer screenings among this population when compared to our catchment area. Muslim women had lower mammography screening rates compared to women in Washington D.C., Virginia, and Maryland; however, cervical cancer screening rates among Muslim women were comparable to screening rates in all three states. Muslim men had higher rates of prostate cancer screening than men in Washington D.C., however, lower rates than those in Virginia and Maryland. Colorectal cancer screenings were significantly lower among Muslims when compared to rates in Washington D.C.,Virginia, and Maryland.

The results of the study demonstrated that overall women relied more on their faith beliefs than men when dealing with health issues. We also saw differences in reliance on faith components based on Asian and Black/African American Muslims, the two most self-reported races. Overall, those who did not get screened for breast, cervical, and colorectal cancer relied more on all three faith components as opposed to those who did get screened for these cancers. An interesting observation in this analysis showed that men who got screened for prostate cancer were more likely to seek guidance from an imam or spiritual advisor when dealing with health issues, compared to men who did not get screened; however, men who did not get screened for prostate cancer were more likely to rely on natural and cultural remedies instead of going to see a doctor, as opposed to those who did get screened for prostate cancer. Understanding the role religious and spiritual factors play in a Muslim man’s decision to get screened for prostate cancer would be of interest to follow up in focus group discussions. Comparisons between different cancer screenings, gender, and faith beliefs show there is a nuanced relationship between the different variables, which would require qualitative analysis to further understand. This highlights the belief that some Muslims feel spiritual, physical, and mental health each play an instrumental role in health and healing, and these components oftentimes overlap when dealing with health, illness, and health behaviors [8]. Findings from this study emphasize the need to further explore how religion, race, and gender intersect and impact faith beliefs and health behaviors across different groups in a diverse American Muslim population.

The present study shows that cancer screening behaviors may be related to certain faith and spiritual beliefs, suggesting that certain religious practices, as well as religious leaders, may help to influence these behaviors. Similarly, literature also suggests cultural and religious beliefs can influence health-seeking and cancer-screening behaviors among different religious, racial, and ethnic subgroups [21–23]. The relationship between Islamic beliefs and health concerns among Muslim Americans may play both a supporting and a hindering role in health behaviors among Muslims. However, there are limited studies that have examined the mechanisms through which these beliefs impact health behaviors, and, more specifically, the role of cancer screening [24]. A thorough examination of this relationship would offer great insights into how religious beliefs impact preventive health and cancer screening behaviors.

Findings from this study suggest a role for the mosque as an institution for health promotion and cancer screening. The majority of participants were in favor of having health initiatives, such as nutrition counseling, physical activity interventions, and cancer screening, treatment, and survivorship education sessions, inside their mosques. Encouraging health-promoting behaviors within a religious lens and framework could be an effective health intervention strategy. Imams, who hold an important role in their communities that include delivering sermons, leading prayer services, providing spiritual support, as well as serving as moral and social counselors [25], may also hold an important role in community health. Specifically, imams may be able to use the faith-based perspective to provide messaging around cancer education and cancer screening to their respective communities. In a previous study of imams, these religious leaders were highly supportive of incorporating cancer screening messaging into faith-based messaging in the form of sermons, individual conversations, and other dialogue [25, 26]. Therefore, the need to further develop and sustain mosque partnerships to build community capacity for health promotion is crucial. Our study has included these key community members from the start of our needs assessment, and we aim to continue working with mosque leadership to tailor outreach and education to fit the needs of this community. Mosques provide a space for religious practice, as well as social and community engagement.

### Limitations of the Study

Our findings can be interpreted in light of several limitations. This study included a convenience sample of 203 participants in four mosques, which may not be representative of Muslims in the Washington D.C. metropolitan area. Thus, our reported screening rates should not be generalized to the entire population of Muslims in the D.C., Maryland, and Virginia areas.

Few studies have assessed the relationship between cultural and religious beliefs and health behaviors. While a question on the survey was asked about the role of cultural remedies when dealing with sickness, our sample size and survey format did not allow us to differentiate between cultural and religious beliefs within Muslim populations, therefore limiting our understanding of what role cultural backgrounds may play in decision making for cancer screening. Additionally, while Muslims of Arab and Middle Eastern descent comprise one of the largest groups in the USA, our survey respondent area comprised of mainly African American and South Asian Muslims, excluding other large, representative groups. Therefore, we cannot generalize the results of this study to all the races and ethnicities of Muslims in the USA. Moreover, in our assessment of screening practices for colorectal cancer, we did not ask about other screening methods for colon cancer, apart from colonoscopy, such as a fecal occult blood test (FOBT) or DNA testing of the stool, which would consider participants to be compliant with respect to colorectal cancer screening. Only asking about a colonoscopy may have prevented participants from disclosing that they did indeed receive a screening for colorectal cancer.

## Conclusions

Findings from our study fill an important gap in the literature about the health education needs, cancer screening practices, and faith beliefs in relation to health behaviors in the Muslim population in the Washington D.C. metropolitan area. Future directions of this initiative would need to involve doing a larger survey with more mosques in the Washington D.C., Virginia, and Maryland areas to better understand how we can offer cancer screening services to this population. Next steps would also include in-depth interviews with Muslims concerning their attitudes and beliefs on cancer screening to further inform the development of educational programming in spaces of worship, in hopes of increasing knowledge and receipt of screening in this population. Additionally, future studies should further explore faith leaders’ knowledge of cancer and cancer screening guidelines and solicit their feedback on how to best contribute to improving health literacy and cancer screening rates in their communities. Such investigations will be key to identifying mechanisms to incorporate health promotion messaging, education, and other health initiatives into mosques in an effective culturally and religiously sensitive way. Connecting how faith-based components of this needs assessment can promote positive health behaviors is crucial to improving the cancer screening rates and overall health in this population.

## Data Availability

The data collected for this study are not openly available due to reasons of cultural sensitivity and are available from the corresponding author upon reasonable request. Data are currently stored in a secure electronic environment at Georgetown University.
